# Editorial: Microorganisms for enhanced carbon sequestration and biogeochemical cycling during soil remediation

**DOI:** 10.3389/fmicb.2026.2012085

**Published:** 2026-10-14

**Authors:** Shu Wang, Yanling Wang, Yuxuan Wan, Belay Tafa Oba

**Affiliations:** 1 PowerChina Northwest Engineering Corporation Limited, Xi'an, China; 2 School of Civil Engineering and Architecture, East China Jiaotong University, Nanchang, China; 3 School of Environmental Science and Engineering, TianGong University, Tianjin, China; 4 Department of Chemistry, College of Natural and Computational sciences, Arba Minch University, Arba Minch, Ethiopia

**Keywords:** carbon emissions, material exchange, microbial consortia, microbial diversity, soil remediation

Microorganisms underpin the stability and multifunctionality of terrestrial ecosystems by driving the coupled biogeochemical cycles of carbon (C), nitrogen (N), and other essential elements. In the context of soil remediation, the mediating role of microorganisms is critical. The decline in microbial diversity has been identified as a key driver of imbalances in farmland soil nutrient cycling. Consequently, restoring microbial diversity or modulating community structures through technological interventions to rebuild soil functionality represents a highly promising research direction. Retention of soil organic carbon can significantly enhance microbial diversity, optimize nitrogen and phosphorus cycling efficiency, and thereby facilitate soil ecosystem restoration. Notably, terrestrial ecosystems offset approximately 30% of anthropogenic carbon emissions. Enhancing soil carbon sequestration capacity by regulating microbial functions not only improves saline-alkali soil structure but also synergizes with carbon neutrality and peak carbon emissions targets.

This Research Topic was established to investigate microbial regulatory mechanisms for synergistically enhancing farmland ecosystem functions, notably addressing soil fertility degradation caused by salinization and nutrient imbalance. By investigating the functional regulatory networks of the microbiome, this Research Topic prioritizes the analysis of activity patterns in key metabolic enzyme systems to elucidate microbially mediated redistribution mechanisms of carbon and nitrogen metabolic fluxes. Through the construction of a functional microbial marker database and the development of detection-driven multi-omics tracing technologies, the research presented here establishes microbial intervention thresholds that simultaneously enhance soil organic matter content and ensure stable crop productivity.

A central theme emerging across this Research Topic is the impact of land management and physical disturbances on microbial community assembly and soil organic carbon (SOC) dynamics. Understanding how topography and physical barriers shape microbial function is essential for ecological security. In nutrient-poor sloping farmlands, it identifies a critical slope threshold (30–45°) where dissolved organic carbon (DOC) availability stimulates microbial synthesis of oxidative enzymes, potentially intensifying long-term SOC depletion (Liu Y. et al.). Similarly, physical alterations to the soil surface, such as cement hardening in industrial areas, significantly reshape microbial diversity and network stability. Hao et al. reveals that while cement hardening increases network complexity, it reduces fungal robustness and enriches taxa involved in respiration while suppressing xenobiotics catabolism, offering new insights for the ecological management of industrial lands.

Equally robust insights emerge from studies focusing on sustainable soil utilization and waste recycling. The sustainable repurposing of dredged sediment is a critical challenge in inland waterway construction. Yaofei et al. demonstrates that amending dredged sediment with composted pig manure, sawdust, and biological agents creates a viable planting soil that sustains higher nutrient availability and microbial activity compared to local topsoil. In the context of contamination, valley-type landfills present complex multi-factor interactions. Xiao et al. elucidates that soil pH and Pb are key regulators of bacterial communities in these environments, with leachate exposure significantly altering the abundance of phyla such as Proteobacteria and Acidobacteriota, thereby influencing C/N/S cycling functions.

A further conceptual advance of this Research Topic is the detailed mechanistic understanding of agricultural practices-specifically straw return and fertilization-on microbial succession and greenhouse gas (GHG) emissions. Long-term straw returning practices drive significant shifts in fungal community composition. Liu R.-Z. et al. highlights that while fungal diversity peaks in the mid-term (years 4–5), different tillage practices (deep plowing vs. no-tillage) lead to distinct functional differentiations, with deep plowing favoring symbiotic guilds and no-tillage favoring pathogen-associated signals. Regarding nitrogen management, Meng et al. utilizes metagenomics to clarify how N fertilizer rates regulate the abundance of functional genes involved in C and N cycling. The study identifies that reasonable nitrogen application (N240) optimizes bacterial diversity and regulates genes related to denitrification and methane metabolism, providing a theoretical basis for emission mitigation strategies.

Finally, this Research Topic addresses the complex biotic interactions and stabilization mechanisms that drive soil multifunctionality. In subtropical karst plantations, Kang et al. finds that while microbial β-diversity positively influences soil multifunctionality (SMF), the complexity of bacterial-fungal-protistan networks can have a negative impact, with sensitive fungal and protistan taxa playing a crucial role. On the mechanistic side of carbon sequestration, Han et al. reviews the dual role of ectomycorrhizal (ECM) fungi, which simultaneously promote C accrual through necromass inputs and drive C loss via enzymatic priming, highlighting the need to integrate ECM traits into ecosystem models. Complementing this, Shen et al. shows that high-carbon bio-organic fertilizers reshape soil aggregate structure and enrich specific bacterial genera, promoting the physical protection of labile carbon within macroaggregates in red soils.

Based on the synthetic insights from this Research Topic, we highlight high-priority directions for future research. First, more integrative models are needed to explicitly link microbial physiological processes and functional traits to ecosystem C–N dynamics under varying physical disturbances. Second, future studies must better address the interactive effects of multiple co-occurring stressors-such as heavy metals, slope erosion, and nutrient imbalance-on microbial functional stability. Third, trait-based microbial indicators should be developed for monitoring soil health and restoration effectiveness across different land-use types, from industrial zones to karst forests.

In summary, the Research Topic establishes microbial-driven remediation and nutrient cycling as a central pillar of terrestrial ecosystem functional ecology. By integrating microbial physiology, functional traits, and community dynamics, the compiled studies clarify how microorganisms regulate ecosystem function and respond to global environmental change. The perspectives presented here provide new quantitative and conceptual tools for predicting ecosystem responses, restoring degraded lands, and enhancing climate resilience.

